# Evolution of dispersal capacities during range expansion: size and behaviour matter in an arthropod invading the sub-Antarctic Kerguelen archipelago

**DOI:** 10.1098/rspb.2025.1136

**Published:** 2025-07-02

**Authors:** David Renault, Yannick Rantier, Peter Convey, Benjamin Bergerot

**Affiliations:** ^1^ ECOBIO (Ecosystèmes, Biodiversité, Evolution), UMR 6553, University of Rennes, CNRS, Rennes 35042, France; ^2^ Biological Sciences, British Antarctic Survey, Cambridge CB3 0ET, UK; ^3^ Department of Zoology, University of Johannesburg, Auckland Park, South Africa; ^4^ Biodiversity of Antarctic and Sub-Antarctic Ecosystems (BASE), Millennium Institute, Santiago, Chile

**Keywords:** dispersal, phosphoglucose isomerase, sinuosity, dispersal path, spatial sorting, biological invasion

## Abstract

The flexibility of movement behaviour was investigated in the non-native carabid beetle, *Merizodus soledadinus*, by comparing individuals from well-established populations (residents) to those at the invasion front (dispersers) in the sub-Antarctic Kerguelen archipelago. Morphology-dispersal covariation was tested by examining how morphology translates into dispersal efficiency and endurance by implementing in-field measurements of dispersal path, sinuosity and tortuosity. The activities of the enzymes phosphoglucose isomerase and pyruvate kinase were also measured to compare putative physiological changes associated with dispersal and residence. In general, the results obtained confirmed that insects from more recently invaded habitats were characterized by larger body sizes. Furthermore, adults of *M. soledadinus* sampled at the invasion front were also characterized by higher locomotor performance, as indicated by longer dispersal paths with less directional changes than their relatives from the founder population. Finally, the activity of the enzyme phosphoglucose isomerase, a powerful estimator of individual dispersal capacity, was higher in insects from invasion fronts. Taken together, our findings consistently indicated that beetles collected from populations at invasion front with the shortest residence times were characterized by enhanced dispersal performance, probably explaining the accelerating range expansion of the species.

## 1. Introduction

Anthropogenically facilitated transfers of species beyond their native distributions are accelerating across the globe [1], with increasing frequencies of introduction and successful establishment events in novel regions [2,3]. Those populations that are established can subsequently proliferate, disperse and, in turn, invade further previously unoccupied areas [4]. During the range expansion of such introduced species, size and behaviour (exploration, movement and dispersal abilities) are key factors shaping their developing new distributions [4,5].

Passive movement and active dispersal are particularly important drivers of geographic spread and biological invasion [6], and selection for dispersive phenotypes along invasion gradients has increasingly been reported [7–9]. Assortative mating among dispersers and low density in dispersal front populations, which lower the requirements for competitive ability [10,11], further enhance the evolution of dispersal-favouring characteristics [12]. In addition, the spatial distribution of invasive species generally follows the common leptokurtic movement found in most animals, including insects. This pattern is characterized by a large number of individuals residing in a given locality (termed ‘residents’), and a smaller number of individuals that disperse effectively (termed ‘dispersers’) [13,14]. Together, these processes lead to spatial selection, resulting in a clear distinction between resident and colonization front populations owing to strong directional selection on dispersive phenotypes at the range edges [15].

Dispersal promotion in range-expanding species can be correlated with specific life-history traits [5,6]. In insects, body size has often been considered an important morphological trait associated with successful range expansion, due to the high energetic demand required by dispersal and the potential for greater energy/resource storage in larger insects [6]. In the butterfly, *Anartia fatima*, dispersal ability is associated with increased thoracic mass allocation [16]. In the carabid beetle, *Carabus hortensis*, the body size of males, but not females, was greater in specimens collected at the range edge [17]. Finally, Therry *et al*. [18] reported that, in the dainty damselfly, *Coenagrion scitulum*, the ratio of flight muscle to fatless body mass was greater at the range limit. However, being larger does not in itself automatically guarantee successful dispersal, and various other traits may improve dispersal and decrease the energetic costs incurred during movement. For instance, a physiological dispersal syndrome was reported in the mite, *Tetranychus urticae* [19], with mites from more dispersive populations characterized by lower amino acid concentrations [14,20].

In terms of behavioural traits, locomotor activity often correlates with dispersal, as reported in the Glanville fritillary butterfly, *Melitaea cinxia* [21], the red flour beetle, *Tribolium castaneum* [22] and the fruit fly, *Drosophila melanogaster* [23]. However, although various behavioural traits have been characterized in the phenotypes of dispersing organisms, such as exploratory tendency, aggressiveness or navigational capability [24], they have been little considered in insects [14]. To date, insect dispersal pathways have generally been derived through modelling techniques, where the dispersal route is considered as a combination of several path sequences, with a straight line connecting two points [25], and there are fewer studies based on direct observations in invasion contexts.

How insects move during dispersal in a biological invasion context has largely been neglected, most likely because *in situ* observations have remained very difficult to obtain. For example, loggers allowing direct recording of the location of individuals are currently too large to attach to most insects (but see studies carried out on larger insects such as bees [26] or beetles [27]). A sufficient number of insects must also be monitored as trajectories may differ among individuals, with dispersal paths either being more direct in dispersing morphs [28], or more tortuous in non-dispersing, with cascading consequences for dispersal costs and distances. Consistent with this suggestion, Klarevas-Irby *et al*. [29] demonstrated that straighter paths (as a proxy for spatial efficiency) and greater dispersal velocity (proxy for time efficiency) were apparent in dispersing rather than resident vulturine guinea fowl (*Acryllium vulturinum*), contributing to a reduction in the energetic costs of dispersal.

The physiological drivers associated with enhanced movement and dispersal abilities have been reviewed by Goossens *et al*. [30]. In the aquatic crustacean, *Daphnia* sp., the frequency of the phosphoglucose isomerase gene was higher in newly established populations, and individuals were characterized by higher metabolic rates and increased dispersal propensity [31]. Since confirmation of the contribution of phosphoglucose isomerase to the dispersal ability of Glanville fritillary butterflies [32], several studies have examined allelic variations in its encoding gene in association with flight performance (see [33]). Several other genes may be equally important contributors to dispersal efficiency [34], but physiological implications in terms of enzyme activities or metabolic fluxes remain poorly explored. Overall, there is currently poor knowledge of the physiology of movement [30], and particularly that of invasive species expanding their ranges.

In this study, we investigated, first, how phenotypic morphological variation in individuals from the colonization front and resident populations are related to movement and, second, the physiological changes potentially associated with movement. To do this, we studied the flexibility of movement behaviour by comparing individuals of the invading predatory carabid beetle, *Merizodus soledadinus*, from well-established populations ('residents', Port-Couvreux, Port-Elisabeth) to those at the invasion front ('dispersers', Isthme-Bas, Val Studer) in the sub-Antarctic Kerguelen Islands in the southern Indian Ocean. The use of *M. soledadinus* as a model species takes advantage of its well-known 100-year invasion history in this archipelago [35]. We have previously reported that larger beetles are more likely to reach more distant locations during colonization events [9]. Thus, we hypothesize that a range of morphometric traits (e.g. pronotum and elytron sizes as metrics of body size, last abdominal sternite (STER) as a measure of the size of genitalia and femur length as a proxy of movement ability) of beetle populations will increase during the range expansion giving increased dispersal abilities, and that larger individuals with longer legs (femur) are more likely to reach more distant locations during colonization events. Morphology-dispersal covariation was tested by examining how morphology translates into dispersal efficiency and endurance. Assuming that the consistency of this hypothesized dispersal proxy was confirmed, its quantitative differentiation from long-term time series along an invasion timeline was then considered. Physiological measurements were used to compare putative physiological changes associated with dispersal and residence. This multi-scale phenotyping (i.e. dispersal behaviour, morphometrics, physiological tests) contributes to understanding how disperser and resident performance may change in different situations, and how variation is maintained in the different populations.

## 2. Material and methods

### (a) Insect sampling

Most species of Carabidae are difficult to rear, with larvae having very low survival rates under controlled conditions. In the present study, field-collected adult beetles of unknown ages were used, thus representing the range of mobility phenotype of each studied population. Adults of *M. soledadinus* were manually collected in the field in December 2018 from four different locations in the Kerguelen Islands: Port-Couvreux 69°41′23.06″ E, 49°16′50.08″ S), Port-Elisabeth (69°51′48.68″ E, 49°13′38.22″ S), Isthme-Bas (70°19′15.56″ E, 49°21′9.52″ S) and Val Studer (70°02′53.83″ E, 49°17′16.45″ S) (electronic supplementary material, S1). The beetle was introduced at Port-Couvreux in 1913, and subsequently dispersed from this founder population, reaching Port-Elisabeth in the 1970s, Isthme-Bas in 2010−2011 and Val Studer in 2012. Port-Couvreux and Port-Elisabeth were considered as well-established populations mainly composed of ‘resident’ insects, while Isthme-Bas and Val Studer were considered as ‘recent’ populations comprising more disperser insects.

At each sampling point, GPS coordinates were recorded and a total of 100 *M*. *soledadinus* adults were collected. Upon collection, insects were brought back to the research station (Port-aux-Français) on the Kerguelen archipelago, where they were maintained at 8°C in incubators (MIR154 Panasonic) and fed with larvae of the native fly, *Anatalanta aptera*. Assessments of in-field movements and dispersal capacities of adult *M. soledadinus* were performed 48 h after collection.

### (b) Real-time tracking of insect dispersal

In-field movements and dispersal of adult *M. soledadinus* collected from the four locations were assessed individually in December 2018 and January 2019 in a common garden experiment carried out on the Kerguelen archipelago. Real-time tracking of individuals (*n* = 14 per population) was performed on the sandy beach of Baie de l’Aurore Australe (70°11′10.50″ E, 49°20′56.51″ S), which is not a representative environment for the locations from which the beetles were collected, thereby providing a novel environment for all assayed insects. Importantly, this location corresponds to habitats where the species occurs naturally in coastal areas of the Kerguelen archipelago, and adults are frequently observed on sand and around stranded debris such as seaweed, where they predate larvae of the native and endemic flies, *Anatalanta aptera* and *Calycopterx moseleyi*, and of the invasive fly, *Fucellia maritima* [35,36]. All experiments were performed at low tide, giving greatest exposed beach area and reduced opportunity for the beetles to quickly reach vegetation cover. To control for potential impacts of photoperiod and temperature, all experiments were performed at the same time of the day, in the absence of wind, and under similar meteorological conditions. For each source sampling location, the same number of individuals was assayed each day in order to minimize potential biases that variation in daily environmental conditions might have on dispersal patterns.

At the start of each observation, one adult *M. soledadinus* was placed on non-trampled sand in the middle of the beach area (Start). Each individual beetle was tracked for a maximum of 35 min; this duration was fixed after preliminary trials in which the median time after which the insect stopped moving occurred after 30−40 min. The dispersal path was continuously traced on the sand with a nail by meticulously following the displacement of the insect. Care was taken by the observer not to shade the beetle, and to mark the route very lightly to avoid the creation of features on the sand surface that the beetle might respond to. In some instances, the tracking ended before the 35 min time limit, when the beetle hid under rocks or debris, or was washed away by wave action. Monitoring also ceased if the individual remained motionless for 10 min. At the end of the observation period, the end of the dispersal path was marked, and the individual was collected and directly preserved in a 2 ml tube containing 1 ml 96% ethanol. For the 14 insects collected from each population, the eight week field deployment period at the Kerguelen archipelago allowed the full dispersal route to be obtained for 12 individuals from each population.

### (c) Characteristics of the dispersal path

A DGPS instrument (Trimble HX6000) was used for recording the start and end points of the dispersal path, and the (Euclidean) distance between these two points was measured. After the end of observation, the route was then re-marked with a nail to improve its visibility on the sand. Photographs (Ricoh GR II equipped with a fixed lens, 28 mm focal length and installed on a telescopic pole) were taken at the highest resolution of the RAW formats). The equipment was connected to a tablet (Panasonic) allowing visualization and validation of the captured images. A grid reference for the standardization of horizontal distance measurements, and a minimum of three PhotoScan testpatterns (for subsequent photogrammetry) were placed before taking each picture. A minimum of 80% overlay was established between each consecutive picture to facilitate the subsequent reconstruction of the dispersal path and photogrammetry (electronic supplementary material, S2). Pictures were taken from an angle as close as possible to perpendicular to the route.

DGPS data were analysed with the Pathfinder Office Pro software. Pictures of the dispersal paths were computed with the Agisoft Metashape software (electronic supplementary material, S2). All information was georeferenced in the WGS84-UTM42S system, and incorporated in a GIS (geographic information system—ArcGIS) file so that a vectorial digitization (point, line and polygon) could be obtained, permitting calculation of the distance covered by each individual and of the sinuosity index.

### (d) Morphological measurements

Morphological measurements were made of all specimens of *M. soledadinus* whose dispersal was tested (*n* = 12 adults for each population), which was increased to *n* = 38 in total for each studied population (*n* = 4). Measurements were carried out as described by Laparie *et al*. [37] and Ouisse *et al*. [38]. In short, the sex of each beetle was first determined under a stereomicroscope (Stemi 305, MAT, trino ESD, Zeiss, Munich, Germany), and pictures of five morphometric traits were taken using a video camera (AxioCam ERc 5 s, ZEISS, Munich, Germany) connected to the stereomicroscope: inter-ocular distance (INTOC), width (PRONOW) and length (PRONOL) of the thorax, length of the right elytrum (ELYT), length of the last abdominal STER and length of the right hind leg (FEMU). The measured morphological traits were used as proxies of body size (INTOC, PRONOW, PRONOL and ELYT), size of the genital apparatus (STER) and movement ability (FEMU). Morphological traits were measured by vectorial layouts with AxioVision software.

### (e) Physiological assays

Physiological assays were carried out to extract and quantify amounts of body proteins and measure the activities of pyruvate kinase and phosphoglucose isomerase. Each specimen of *M. soledadinus* was transferred to a new 2 ml microtube containing 120 μl phosphate buffer and two tungsten beads. Each beetle was homogenized for 1 min 30 s at 25 Hz (Bead beater RetschTM MM301, Retsch GbmH, Haan, Germany). After centrifuging at 4000*g*, 4°C, for 10 min, 5 μl of the supernatant was pipetted into the well of a microplate. Then, 235 μl of Bradford’s reagent was added to each well, and the microplate was homogenized and incubated at room temperature for 20 min before absorbance was read at 595 nm [39]. Calibration curves were generated using bovine serum albumin (Thermo Scientific, Waltham, MA, USA) diluted in phosphate buffer and used for calculating the protein content of each sample.

The remaining 115 μl of each sample were used for measuring activities of pyruvate kinase (PK, EC 2.7.1.40) and phosphoglucose isomerase (PGI, EC 5.3.1.9). For each assay, the recommended protocols of the manufacturers were followed. For pyruvate kinase (Assay kit MAK072, Sigma-Aldrich, St-Louis, MO, USA), 15 µl supernatant from each sample was mixed with 35 µl buffer and 50 µl reaction mix, and the absorbance was read every 2 min for 20 min at 570 nm. Pyruvate standards were prepared for the calibration curves. For phosphoglucose isomerase (Assay kit MAK103, Sigma-Aldrich, St Louis, MO, USA), 5 µl supernatant of each sample was mixed with 45 µl of phosphate buffer and 50 µl reaction mix, and the plate was incubated at room temperature for 5 min before the absorbance was read every 2 min for 10 min. NADH standards were prepared for the calibration curves. Enzyme activities are presented as milliunit ml^−1^; as body size and mass may differ between individual insects, the body protein amount was also used as a proxy for insect mass and activities are therefore also presented in unit mg^−1^ of body proteins.

### (f) Statistical analyses

The movement trajectories of the 48 recorded individuals were digitized (electronic supplementary material, S3) at 5 cm resolution and different indices were calculated to characterize the movement of each individual using the R package ‘trajectories’ [40]. The path length was calculated for each individual. Five indices characterizing movement based on the individual trajectories were used: (i) The sinuosity index ‘S2’, which calculates the sinuosity of a constant step length trajectory [41]. This is a corrected version of the sinuosity index defined in Bovet and Benhamou [42], which is suitable for a wider range of turning angle distributions. (ii) The maximum expected displacement ‘TE’ which is a single-value measure of straightness [43]. TE is a dimensionless, scale-independent measure of the maximum possible expected displacement. Values closer to 0 are more sinuous, while larger values (approaching infinity) are straighter. (iii) The straightness index ‘TS’ of a trajectory calculated using the formula D/L, where D is the direct distance between the first and last points in the trajectory, and L is the path length travelled [44]. The straightness index is considered to be a reliable measure of the efficiency of a directed walk, but is inapplicable to random trajectories [41]. (iv) The mean time variation of directional change ‘DC’ and (v) the standard deviation for the time variation of directional change ‘SDDC’ of a trajectory [45]. Directional change is defined as the angular change (in degrees) between any two points in the trajectory, divided by the time difference between the two points.

To compare the movement indices and morphological and physiological characteristics of individuals based on their origin and hypothesized mobility phenotypes (residents: insects from Port-Couvreux and Port-Elisabeth; dispersers: insects from Isthme-Bas and Val Studer), one-way analyses of variance (ANOVA) were performed. Where significant, Tukey honestly significant difference (HSD) *post hoc* tests were used to identify significant differences between locations of origin.

After data exploration (range of correlation values in electronic supplementary material, S4), we retained four uncorrelated variables from the initial set of measured variables, these being two morphological variables (STER and FEMU) and two physiological variables (activities of the enzymes pyruvate kinase and phosphoglucose isomerase). These variables, as well as possible interactions with origin and sex, were taken into account using a general linear model (GLM) to explain movement indices. Except for TE (which follow a Poisson distribution), GLMs were constructed assuming Gaussian distributions and we used a backward selection process based on the Akaike information criterion [46] to select the final model. The general starting model included movement indices as dependent variables with STER, FEMU, activities of pyruvate kinase and phosphoglucose isomerase and potential interactions with the sex and the origin as explanatory variables. Analyses of variance of the GLMs were made using a type 3 ANOVA and associated *p*-values were calculated. Adjusted *D-*squared calculations were also provided to give a comparable percentage of deviance for each model because one model (TE) did not follow a Gaussian distribution [47]. All data analyses were performed using R software [48].

## 3. Results

### (a) Movement Indices

**Figure 1.**
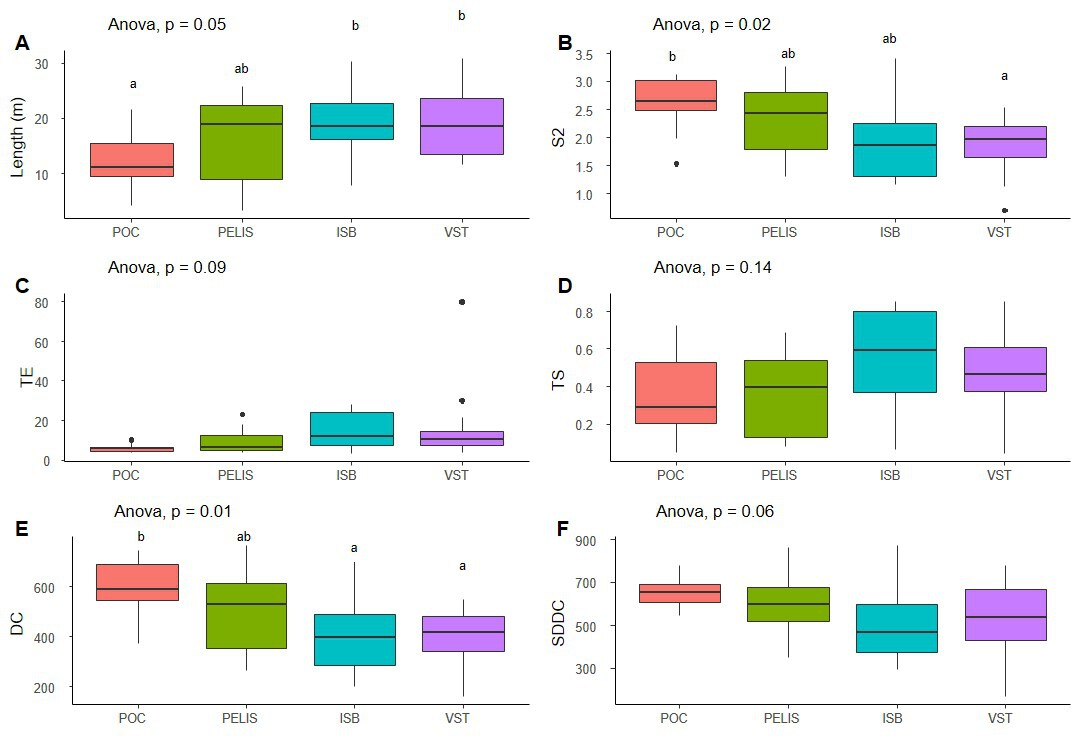
Mean values ( ±s.e.) of (A) path length (Length), (B) the corrected sinuosity index (S2), (C) the maximum expected displacement (TE), (D) the straightness index (TS) of a trajectory, (E) the mean time variation of directional change (DC) and (F) the standard deviation for the time variation of directional change (SDDC) of a trajectory in each of the four locations (‘residents’: Port-Couvreux and Port-Elisabeth; ‘dispersers’: Isthme-Bas and Val Studer). The significance of ANOVA results is indicated (*n* = 48 individual beetles, 12 per location). Letters indicate significant differences between locations. ISB, Isthme-Bas; PELIS, Port-Elisabeth; POC, Port-Couvreux; VST, Val Studer.

Based on the 48 individuals observed, the mean path length (length, figure 1A) was significantly greater for those originating from the more recently colonized localities of Isthme-Bas and Val Studer compared with Port-Couvreux (founder population). Individuals from Port-Couvreux showed significantly more sinuous paths than those from Val Studer (electronic supplementary material, S4, figure 1B) and with more marked changes in direction (figure 1E) than those from both Val Studer and Isthme-Bas. For most movement indices, individuals from Port-Elisabeth were characterized by an intermediate pattern.

### (b) Morphological variables

**Figure 2.**
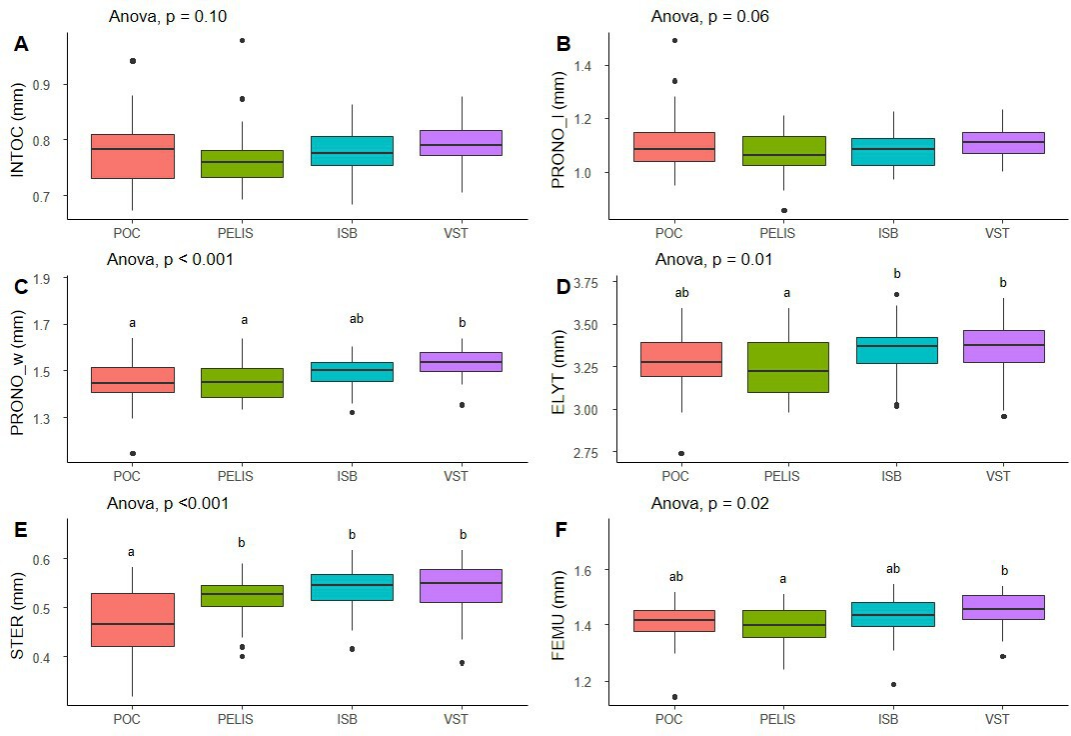
Mean values ( ±s.e.) of (A) inter-ocular distance (INTOC), (B) pronotum length (PRONO_l), (C) pronotum width (PRONO_w), (D) elytra length index (ELYT), (E) sternite length (STER) and (F) femur length (FEMU) in each of the four locations (‘residents’: Port-Couvreux and Port-Elisabeth; ‘dispersers’: Isthme-Bas and Val Studer). The significance of ANOVA results is indicated (*n* = 152 individual beetles). Letters indicate significant differences between locations. ISB, Isthme-Bas; PELIS, Port-Elisabeth; POC, Port-Couvreux; VST, Val Studer.

Based on measurements of a total of 152 individuals, the pronotum width (PRONO_w, figure 2C) was significantly greater in Val Studer than Port-Couvreux and Port-Elisabeth. The size of the elytron (ELYT, figure 2D) was longer in Isthme-Bas and Val Studer than in Port-Elisabeth. The size of the sternum (STER, figure 2E) and femur (FEMU, figure 2F) was significantly greater in individuals from Val Studer than Port-Couvreux.

### (c) Physiological variables

**Figure 3.**
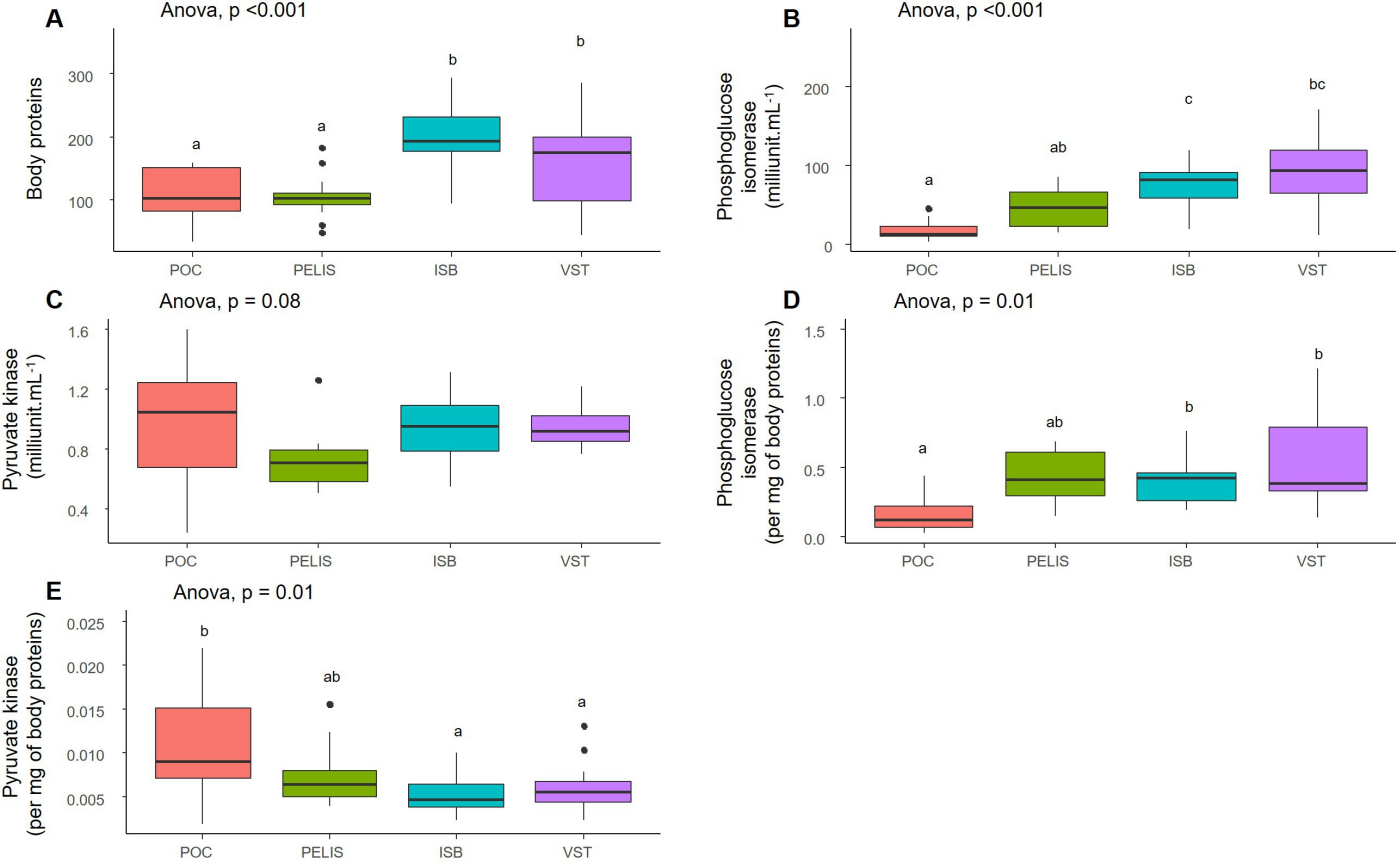
Mean values ( ±s.e.) of (A) body protein content, (B) phosphoglucose isomerase activity (PGI), (C) pyruvate kinase activity (PYRU), (D) Phosphoglucose isomerase activity per mg of body protein (PGI, unit mg^−1^ body proteins), and (E) pyruvate kinase activity per mg of body protein (PYRU, unit mg^−1^ body proteins) in each of the four locations (‘residents’: Port-Couvreux and Port-Elisabeth; ‘dispersers’: Isthme-Bas and Val Studer). The significance of ANOVA results is indicated (*n* = 56 individual beetles). Letters indicate significant differences between locations. ISB, Isthme-Bas; PELIS, Port-Elisabeth; POC, Port-Couvreux; VST, Val Studer.

Based on measurements carried out on 56 individuals, the quantity of body proteins was significantly lower in individuals from Port-Couvreux and Port-Elisabeth than from Isthme-Bas and Val Studer (figure 3A). Phosphoglucose isomerase activity, expressed in milliunit ml^−1^ (figure 3B) or mg^−1^ of body proteins (figure 3D) was significantly lower in individuals from Port-Couvreux than Isthme-Bas and Val Studer. Conversely, pyruvate kinase activity mg^−1^ of body proteins (figure 3E) activity was significantly higher in individuals from Port-Couvreux than Val Studer and Isthme-Bas. For most physiological variables, insects from Port-Elisabeth were characterized by intermediate values.

### (d) Models

The adjusted *D*-squared ranged from 0.18 to 0.51 (table 1). With the exception of the maximum expected displacement model (TE, adjusted *D*-squared value = 0.18), the adjusted *D*-squared values were all above 0.35 (the standard deviation of the time variation of the direction change of a trajectory model, SDDC). Location was identified as an important variable, as it was significant in five of six models. Although the model for path length was not significant, its *p*-value was <0.1 (table 1). The two variables that stood out the most in the models were femur size and the interaction between location and pyruvate kinase activity (milliunit ml^−1^) (significant in four of six models). These variables explained the maximum expected displacement (TE), the straightness index of a trajectory (TS), the mean time variation of the direction change (DC) and the standard deviation of the time variation of the direction change of a trajectory (SDDC). Finally, three of six models identified pyruvate kinase activity (milliunit ml^−1^) as significant, namely, the maximum expected displacement (TE), the straightness index of a trajectory (TS) and the mean time variation of the direction change (DC). Three of the six models identified the interaction between location and femur size, namely, the maximum expected displacement (TE), the straightness index of a trajectory (TS) and the standard deviation of the time variation of the direction change of a trajectory (SDDC).

**Table 1.** General linear model (GLM) results explaining movement indices (S2, the corrected sinuosity index; TE, the maximum expected displacement, TS, the straightness index of a trajectory; DC, the mean time variation of directional change, SDDC, the standard deviation for the time variation of directional change of a trajectory) according to location (LOC), sex (SEX), morphological variables (FEMU, femur length, STER, sternite length), physiological variables (activity of the enzymes pyruvate kinase [PYRU] and phosphoglucose isomerase [PGI] in milliunit ml^−1^) and their interactions. ***p < 0.001; **p < 0.01; *p < 0.05; ^•^*p* < 0.1; —, variable not retained in the final model following AIC selection procedure.

	degree of freedom	path length	S2	TE	TS	DC	SDDC
*family*		Gaussian	Gaussian	Poisson	Gaussian	Gaussian	Gaussian
LOC	3	^•^	*	***	**	**	**
SEX	1		*		**		
FEMU	1			*	**	**	**
STER	1		^•^				—
PGI	1			*			**
PYRU	1			*	*	**	
LOC:SEX	3	*			—	—	—
SEX:FEMU	1	—			**	—	—
SEX:STER	1		^•^			—	—
LOC:FEMU	3	^•^		***	—	***	**
LOC:STER	3			***	*		—
LOC:PGI	3	^•^		***	—	^•^	*
LOC:PYRU	3	^•^	^•^	***	***	*	*
adjusted *D* squared		0.40	0.45	0.18	0.41	0.51	0.35

## 4. Discussion

In invasion processes, range expansion by non-native organisms is partly driven by dispersal capacities, with those individuals with the highest dispersal performance being expected to be capable of dispersing further, additionally setting the speed at which novel suitable habitats can be invaded [49]. Among the mechanisms potentially contributing to invasion success, it is often expected that non-native organisms perform better in their invaded ranges in comparison with home ranges [50], with phenotypic differences being further increased along invasion gradients [14]. Spatial sorting of populations may take place during the range expansion, one result of which can be the selection of dispersing phenotypes at the invasion front. However, the magnitude of this process is likely to be impeded by a range of life-history trade-offs and costs of plasticity that may either facilitate or limit invasion success [51,52]. Variations from core to edge populations in competition and population density may further differentially alter trade-offs among functional traits. However, there are few ecologically relevant studies available examining movement capabilities, and thus dispersal performance, in insects from non-native populations along invasion gradients. In this study, building on previous investigations using the invasion of the Kerguelen archipelago by the beetle *M. soledadinus* as a model system, we compared movement and physiological characteristics among beetle populations sampled from older (Port-Couvreux, Port-Elisabeth) and more recently established (Isthme-Bas, Val Studer) populations in the archipelago.

In general, we found that insects from more recently invaded habitats (Isthme-Bas and Val Studer) were characterized by larger body sizes (pronotum width, elytron length), thus confirming the morphological gradient previously reported by Laparie *et al*. [9,37]. Pronotum width and STER length were significantly smaller in individuals from Port-Couvreux (the founder population) in comparison to Val Studer, and a tendency was also apparent for elytron and femur lengths (a proxy for dispersal capacity) to be smaller in individuals from Port-Couvreux. As adults of *M. soledadinus* are voracious predators and quickly establish large populations in invaded habitats [35], size differences along invasion gradients may be partially driven by differential accessibility to trophic resources across the studied localities. At the invasion front, colonists would benefit from a larger—potentially unlimited—pool of preferred prey, supporting the production of larger offspring. However, this hypothesis cannot solely explain the body size pattern observed here, as the pattern remains apparent several years after the establishment of populations of *M. soledadinus* [9,37] when the quality of the trophic resource has already declined. Furthermore, this pattern has also been reported for non-predatory insects [53].

Empirical studies have reported longer legs, larger thoracic muscles, increased muscular efficiency, as well as greater starvation tolerance and body energy stores [31,54–57] in specimens collected at invasion fronts. Insects with larger body sizes have higher dispersal efficiency and capabilities to reach distant habitats, and assortative mating of these individuals further contributes to maintaining higher body sizes, as demonstrated experimentally in the damselfly, *Coenagrion scitulum* [58]. However, body size itself may not always be associated with dispersal capacity, as reported in red flour beetles for which a part of the body only involved in movement (relative leg length) was related with movement ability [59]. In the bean beetle, *Callosobruchus maculatus*, Ochocki and Miller [60] experimentally demonstrated the rapid evolution of dispersal ability in insects from spatially sorted populations. The promotion of dispersal traits at the invasion front has also been highlighted in the cane toad as it has rapidly invaded the north-east coast of Australia [15,55], suggesting that this pattern can be repeated across diverse animal taxa. Conversely, in longer established populations, the higher population density and the qualitative and quantitative decline of food resources increase intra-specific competition. This should favour selection for the competitive abilities of individuals, such as the production of more eggs by females (to counteract competition-induced mortality of juveniles) and more rapid juvenile developmental rates, both factors that can lead to the development of adults with smaller body sizes [61]. Core populations of *M. soledadinus* are often close to their carrying capacity, meaning that intra-specific competition is high. Our findings confirmed this observation, with individuals from Port-Couvreux, and to a lesser extent Port-Elisabeth, being generally characterized by smaller body sizes (pronotum and elytron sizes), dispersal features (femur) and size of the genital apparatus (STER). In those well-established populations, further studies are now required to examine if females produce larger numbers of smaller eggs as a result of following a bet-hedging strategy, whereby the higher egg production is assumed to be associated with lower hatching probability in core populations in predatory species such as *M. soledadinus*.

Dispersal performance represents an important factor driving the invasion speed of range-shifting species, and traits supporting dispersal likely coevolve with functional traits influencing fecundity and/or growth rates [21]. Increased movement performance is an explicit advantage for colonizing individuals, and this advantage could be even more significant in successfully reaching more distant locations if dispersing individuals use direct trajectories [62,63]. Here, displacement patterns of adult *M. soledadinus* were explored to further assess the dispersal behaviour of the species. Adults sampled at the invasion front (Val Studer, Isthme-Bas), already had larger body size and were also characterized by higher locomotor performance, as depicted by longer dispersal paths than their relatives from the founder population (Port-Couvreux). While values obtained were often close to those from Port-Couvreux, the dispersal metrics measured in insects from Port-Elisabeth tended to have intermediate values, consistent with their intermediate residence time in comparison to the three other studied populations. Together, these results suggest that dispersal capacities have evolved along the invasion gradient of *M. soledadinus* in the Kerguelen archipelago, with adult beetles from newly established populations characterized by higher dispersal distances as compared with those sampled from longer established populations, and individuals from Port-Elisabeth having intermediate dispersal features. Ouisse [64] previously reported that specimens of *M. soledadinus* from range margin populations exhibited greater locomotor activity under controlled conditions. These findings are consistent with the available literature reporting the existence of a correlation between dispersal ability and body size in various other insect species [65,66]. For instance, in the invasive ladybird, *Harmonia axyridis*, there was a marked increase in the flight speed of insects from the core to the front of the invasion range across two independent sampling transects [56]. Ochocki & Miller [60] also demonstrated experimentally that spatial sorting contributed to a rapid increase in dispersal capacity in the bean beetle and, in the round goby, males collected at the invasion front were characterized by higher dispersal potential [67].

While the propensity of individuals to leave a source patch was not assessed here, our experimental design may have tested for behavioural sorting [68]. As a complement of locomotor activity, we studied the directness of the walking path (sinuosity index, directional change) of adult *M. soledadinus*, both parameters being higher in specimens from the core populations (Port-Couvreux, Port-Elisabeth). By comparing macropterous and brachypterous morphs of *Pyrrhocoris apterus*, Socha & Zemek [28] experimentally demonstrated that the dispersing morph was characterized by a walking path (mean velocity, turn angle, sinuosity) that was distinct from that of their resident relatives, further supporting distinct behaviours among the morphs. At the local scale, the sinuosity of the path measured from individuals of Port-Couvreux and Port-Elisabeth may depict the capacity of individuals to explore the environment and actively search for patchy resources. The distance that an animal moves from its original location over a finite time period depends upon the proportion of time that it spends moving, the rate that it travels when it is moving and the straightness of the path that it follows over time [69]. For individuals at the colonization front, dispersal is very energy consuming and increasing either the proportion of time spent moving or the rate of movement is energetically expensive [70]. Thus, an efficient way for a dispersing individual to increase its net rate of movement is to follow a straighter path. One strategy for limiting this expenditure is to increase the straightness of the path followed, as measured in insects from Isthme-Bas and Val Studer, which could result in a small additional energy cost [70,71]. Thus, the path straightness (i.e. direction of movement) exerts a powerful influence on total distance moved [72,73]. Conversely, the tortuosity of an animal’s path provides insights into its use of space, as highly tortuous paths that thoroughly cover small areas can evolve to maximize search efficiency for a patchy resource [74]. Straighter—less sinuous—dispersal paths may be selected when landscapes must be crossed as rapidly as possible as, for instance, in environments where suitable habitats are distributed in a patchy manner. Finally, it has also been suggested in range-expanding species that straighter displacement lines may evolve at the expense of survival [73]. Even if this aspect was not tested in the present study, it is appropriate to note that Géron *et al*. [57] demonstrated that adult *M. soledadinus* from recently established populations exhibited higher capabilities to resist environmental stress.

The combination of behavioural, physiological and biochemical changes associated with morphological differences along invasion gradients remains largely unexplored in entomological studies. Several studies have suggested the existence of different phenotypes among core and range edge populations [9,56,58,75], but associations between trait combinations contributing to dispersal in invasion front populations remain to be clarified (but see [57]). In our data, the activity of phosphoglucose isomerase (PGI), an important enzyme involved in cellular energetics, was higher in insects from invasion fronts (Isthme-Bas, Val Studer) and lower in the founder population (Port-Couvreux, Port-Elisabeth). Such a correlation of the activity of phosphoglucose isomerase with residence time is relatively novel in the context of biological invasions in insects. We suggest that it supports higher capacity for energy production, and ly enhanced metabolic performance, in individuals of this flightless carabid beetle from invasion fronts. In other insect taxa, the expression and allelic diversity of the gene encoding PGI has been shown to be a powerful estimator of individual dispersal capacity [76,77]. The genetics of insect dispersal (see review by [78]) has been a focus of research in the Glanville fritillary butterfly [33]. In this insect, newly established individuals are characterized by a higher frequency of a specific allele of the Pgi gene and higher metabolic rate during flight [31]. Ouisse [64] reported that the highest level of genetic diversity was measured in adult *M. soledadinus* from Port-Couvreux, with subsequent stepping-stone range expansion. Heterozygosity correlated with residence time, with individuals from Isthme-Bas and Val Studer showing the lowest values while those of Port-Elisabeth showed intermediate genetic variation. Geographic expansion from the single original introduction site of Port-Couvreux progressively selected insects with enhanced dispersal capacities whose small founder populations, as revealed by the lowest heterozygosity in Val Studer [64], may have helped in maintaining higher allelic frequencies for traits supporting dispersal, such as the expression of Pgi. Such findings emphasize the importance of considering physiological parameters in addition to genetic studies in invasion studies, owing to the importance of physiological plasticity as a driver of evolutionary responses in insects.

## 5. Conclusions

Dispersal is a potentially costly behaviour and strategy, both in terms of the risks involved and the energetic investment required. In the current study, we confirmed that invasion front populations of *M. soledadinus* on the sub-Antarctic Kerguelen archipelago were consistently morphologically distinct from founder populations which have been present on the islands for several decades longer. Such differences have been assumed to be associated with increased movement abilities, fueled by larger body stores. Our detailed observations of beetle movement demonstrated that those from populations with shorter residence times (at or close to the invasion front) were characterized by straighter, less tortuous and longer dispersal paths than those sampled from founder populations. The more direct dispersal trajectories exhibited by insects from invasion fronts probably contribute to accelerating invasion dynamics of this species. Conversely, insects from founder populations were characterized by more tortuous movements consistent with exploratory behaviour. Physiological differences were also apparent between invasion front and founder population beetles, with greater activity of phosphoglucose isomerase, an important enzyme involved in cellular energetics.

## Data Availability

Data and scripts are openly available in GitHub at [79]. Supplementary material is available online [80].
